# Early Rehabilitation in Head Injury; Can We Improve the Outcomes?

**DOI:** 10.5812/atr.13665

**Published:** 2013-12-01

**Authors:** Rajiv Singh, Guruprasad Venkateshwara, Julie Batterley, Sarah Bruce

**Affiliations:** 1 Osborn Neurorehabilitation Unit, Northern General Hospital, UK

**Keywords:** Craniocerebral Trauma, Critical Pathways, Healthcare Quality, Physical and Rehabilitation Medicine

## Abstract

**Background:**

The quality of care after head injury is still very variable with a little coordination between different specialties. Acute care dominates, often with little regard to rehabilitation needs.

**Objectives:**

To improve the outcomes of all head injury admissions to hospital, including mild and moderate, by creating a head injury team to supervise a rehabilitation clinical pathway.

**Patients and Methods:**

A head injury team was established to manage the care of all non-neurosurgical admissions with head injury to a large teaching hospital. Apart from inpatient care, the team coordinates various services involved in the care of head injuries, arranged suitable follow-ups, supported relatives and trained healthcare staff on general wards in the treatment of head injured patients. Follow-up clinics at 6 weeks and 6 months were arranged.

**Results:**

In the first three years, the team managed the care of 812 admissions. Mean age was 44.3 years (SD = 24.8) and mean length of hospital stay was 6.1 days (SD = 10.9). Of these individuals, 674 attended for 6 month follow-up with 52.2% having a good outcome on Extended Glasgow outcome score. Patients and their relatives' feedbacks were excellent with an average score of 4.7/5 on overall satisfaction rating. Following presentations at national meetings and elsewhere, other centers in the United Kingdom are now setting up similar pathways.

**Conclusions:**

A dedicated clinical pathway and head injury team can improve the quality of care for all admissions with head injury and enhance the role for rehabilitation medicine input at an early stage.

## 1. Background

The management of head injury demands a wide variety of specialist skills and presents complex problems. Many individuals never seek medical advice or are discharged from accident and emergency departments with no follow-up and there is a high level of unmet need (1). Those with a severe injury are usually admitted to the neurosurgical or orthopaedic wards. But they are lucky if they receive neurological rehabilitation afterwards or ongoing referral for rehabilitation in the community. Furthermore, the management of those with mild or moderate injury is even more variable and patients can receive a wide range of care. Those who are admitted may end up under a number of different specialties; brain injury specialists in rehabilitation medicine are rarely involved at an early stage. There is no coordination of overall care needs and the lack of responsibility leaves patients and families with an unsatisfactory service and hospitals with a clinical governance risk. This situation is common all over the United Kingdom. In order to address this unhappy situation, we introduced an acute brain injury care pathway in Sheffield. The aim was to improve the caring quality for all brain injury admissions, not just those with a severe brain injury. Prior to the introduction of the pathway, head injuries in this region were admitted to different departments depending on their immediate need on admission without any thought to an overall coordination of care. Such specialties included general surgery, orthopaedics, neurosurgery, ENT, care of elderly, A&E beds or could be discharged the same day.

After admission there was no specialist input from a team specialized in brain injury. Patients were often being discharged with little support or regard for social circumstances. Their families were often put under great strain and the lack of coordinated follow up and inconsistencies in quality of care put the hospital at significant clinical governance risk.

The introduction of national policies and standards including the national services framework for long-term neurological conditions (NSF) and National Institute for Clinical Excellence (NICE) head injury guidelines in 2005 (2, 3) created a drive to develop head injury services. It was clear that many of the guideline requirements could be met by an appropriate head injury pathway, coordinated to meet the needs of patients. Since then, there have been further initiatives to improve the quality of major trauma services nationally. This has added to the impetus to improve head injury care (4, 5).

In recent years, many different areas of medical practice have developed clinical pathways using a multidisciplinary team approach to set standards, assess quality of care, measure performance and avoid inconsistencies of care (6-8). Such pathways are attractive because they improve the quality of care and safety for patients and hopefully improve the outcomes (9, 10). However, a literature search using MEDLINE and EMBASE revealed little in the published literature as regards use of such pathways in head injury management. In severe brain injury, a pathway has been shown to improve some patient outcomes and reduce costs (11). A significant study by Fakhry et al. found that the outcomes could be improved but only included severe brain injuries (12). Other studies have shown that a pathway reduced the length of stay but other outcomes were unchanged (13, 14). However the aim in Sheffield was to improve the care of all admitted injuries not just the most severe injuries. There is a growing awareness of the significant risks to even those with moderate and mild brain injury (15) and the development of head injury guidelines has resulted in many such patients being admitted for overnight observation and management (16, 17). The aims of the pathway was to reduce the variations in quality of care for all admissions with TBI, to bring all admissions under a specialist in brain injury and to aid compliance with meeting national recommendations for head injury care. Clinical governance and patient safety are of vital importance and it was hoped that eventually it would be possible to show an improvement in long-term patient outcomes.

## 2. Objectives

To improve the outcomes of all head injury admissions to hospital, including mild and moderate, by creating a head injury team to supervise a rehabilitation clinical pathway.

## 3. Materials and Methods

A taskforce was set up by a group of key brain injury stakeholders. This included local government, community health providers and voluntary sector organisations as well as hospital departments which routinely admit head injury patients. It was important that all TBI patients were included in the pathway. Six beds were set up as the head injury observations unit (HIOU). An acute brain injury team (ABIT) was created based in the rehabilitation medicine department to have the responsibility for the care of patients admitted to these beds. The team consists of a brain injury specialist doctor, a clinical nurse specialist and a brain injury social worker.

All patients with head injury or suspected injuries are admitted to the observation unit. Criteria for admission are based on the NICE guidelines for head injury (3). These are extensive but include abnormal CT scans not admitted to neurosurgery, diminished Glasgow coma score or any unresolved clinical concerns that preclude discharge (e.g. alcohol intoxication). The unit can also take step-down patients from ITU while a management plan is made for ongoing care or management.

The pathway does not specify the exact treatment required in each the parent specialties involved in head injury care. For instance it has not produced protocols for ITU, ENT or neurosurgical management; these protocols remain the responsibility of the relevant departments. The pathway is about coordinating overall care and being responsible for patients who do not fall clearly into specialties such as neurosurgery. Those patients initially admitted to neurosurgery or ITU are again taken up by the brain injury team on discharge from those units.

Each day, the team joined the emergency ward rounds and take over the care of any patients admitted the previous day. Referrals from other units such as care of elderly can be seen and patients either taken over or advice given. The ABIT is therefore a key link between the varying elements of services that are involved in brain injury, including neurosciences, surgery, ENT, general medicine and care of elderly as well as community services. The team provides smooth transitions between services as required and facilitates appropriate follow ups or reviews by relevant specialists and ascribes to the use of a rehabilitation model at an early stage to improve patient service and outcome.

Patients received therapy input from neurorehabilitation staff who have the appropriate skill set and training for the head injury group. The team manages patients’ care needs on the observation unit and if longer term in-patient care is required for brain injury rehabilitation then patients can be transferred to the main neurorehabilitation ward itself. This is useful for those with more severe injury who require a longer stay or for assessing detailed cognitive problems and safety issues for discharge.

Relatives are often forgotten the acute settings (18) have to deal with ill patients on discharge. Caregiver stress is recognized as a significant problem (19-21). Considerable evidence is emerging that interventions directed at family support can be effective (19, 22). The team was active in supporting families to fulfill their role. The social worker has a key role to play the interface with relatives and can point to the resources such as local head injury groups, benefit agencies and several leaflets developed by the group. On discharge, patients are given contact numbers for continuing support and a referral to community brain injury services is made if needed.

## 4. Results

To facilitate the new pathway, a number of operational policies, referral and transfer criteria, discharge checklist and documentation pro forma had to be devised for head injury observation. A head injury follow-up clinic has been set up for all patients including those discharged from emergency department within 24 hours. At the clinic, any on-going problem is identified and appropriate assessments undertaken. It is known that 5% of even mild head injuries have significant disabling symptoms at the first year and the appropriate management of mild TBI can reduce the incidence of these symptoms (23, 24). The aim of the clinic is to reassure patients and treat any persisting symptoms or complications.

A key benefit of the pathway was to educate other health staff as to the significance of head injury and its treatment. Intuitively, the training of staff and increased confidence in dealing with head injury should improve outcomes. However, this is difficult to show the use of appropriate objective outcome measures. A rolling program to train nursing staff, junior doctors and therapists is in place and the profile of head injury management has been raised across the region. Indeed the pathway has been highlighted nationally through the British society of rehabilitation medicine and other professional bodies as a model of excellence. Presentations on one year data at national and international meetings have highlighted the strengths of such a service and other regional units are looking at the pathway in order to try and recreate similar systems elsewhere. The pathway has featured in the local press and the team lectured on various aspects of brain injury extensively. The team acted as advocates for the importance of brain injury services and hopefully will influence future service development.

In the last year, the United Kingdom has followed models of trauma care in other countries, most notably the United States and has set up regional major trauma centers (4, 5). An important part in caring such individuals is the rehabilitation that they receive (25). The resulting development of trauma rehabilitation in the United Kingdom has acted as a fresh impetus to the role of rehabilitation medicine specialists in the acute stages of traumatic injury and the brain injury team has been pivotal in the development of national as well as the local trauma rehabilitation systems.

For those of us who are interested in rehabilitation medicine (RM) as a specialty, the development of head injury and trauma rehabilitation pathways has presented an opportunity for RM to show its value within acute healthcare systems. Traditionally RM is involved at a later stage after injury if indeed at all. We now have an opportunity to make a difference to patients by introducing good rehabilitation principles at the outset of care rather than waiting for referrals from other colleagues at a later stage. We believe strongly that all head injury patients who are discharged from A&E or after overnight stay, should be followed up by a specialist to reduce the incidence of future problems.

For the hospital trust, the problem of overall patient responsibility has been solved. Patients are now under a specialist in brain injury who will coordinate appropriate referrals and care. Decisions are taken and clinical governance is much improved.

The ultimate measure of success would be to show a change in objective outcomes after head injury. Unfortunately there was no previous record of head injury outcome measurement in our hospital until the team was set up and started to collect such data. It is therefore impossible to show a definitive improvement in any such outcome measurments. Furthermore, it is known that head injury data is notoriously poorly coded (26) and there is considerable variation in the measures that different units use (27). The most common measure that is used is the extended Glasgow outcome score (E-GOS) (28). Compared to most other measures, it is relatively quick to administer and has less room for subjective reporting. This is the key outcome that we decided to report. We have reported previously on one year data but numbers were understandably insufficient and many people took time to become aware of the new service (29).

In Table 1 we present data from the first three years of admissions under the pathway. These are patients who returned to the head injury clinic at 6 months follow-up. In this period, there were 812 admissions to the pathway. Of these, 674 attended both the initial clinic and then follow-up at 6 months for evaluation of outcome using the extended Glasgow outcome score (E-GOS). 

**Table 1.** Clinical and Demographic Features of Head Injury Admissions (based on 674 patients out of 812 who reattended at 6 months)

Characteristic	No. (%)	Mean (SD)
**Gender**		
Male	460 (68.3)	
Female	214 (31.7)	
**Severity of injury**		
Mild	239 (35.5)	
Moderate	293 (43.5)	
Severe	142 (21.0)	
**Etiology**		
Assault	114 (16.9)	
Fall	336 (49.9)	
Road Traffic Accident	170 (25.2)	
Work accident	45 (6.7)	
Fits	9 (1.3)	
**Ethnicity**		
White	635 (94.2)	
Other	39 (5.8)	
**Home support**		
Alone	354 (52.5)	
Supported	320 (47.5)	
**Alcohol excess**		
Yes	182 (27.0)	
No	492 (73.0)	
**Warfarin**		
Yes	51 (7.6)	
No	623 (92.4)	
**CT scan findings**		
Nil	246 (36.4)	
Contusions	191 (28.4)	
Intracranial bleed	164 (24.3)	
DAI	73 (10.9)	
**Depressive symptoms**		
Yes	219 (32.4)	
No	455 (67.6)	
**Glasgow outcome score**		
1 - 4	36 (5.4)	
5. Moderate Lower	120 (17.8)	
6. Moderate Upper	166 (24.6)	
7. Good Lower	203 (30.1)	
8. Good Upper	149 (22.1)	
**Age , mean (SD), y**		44.3 (24.8)
**Length of stay, mean (SD), d**		6.1 (10.9)

From Table 1, it is clear that the majority of individuals had a mild or moderate injury while only 21% having a severe TBI. We also found that a considerable number of patients live alone and that depression was common with 32% showing significant depressive symptoms. It is already well known that mood disorders are common after brain injury ( 30 , 31 ). Emotional difficulties magnified in individuals with cognitive and physical impairments and our results highlighted the need to address this issue. The role of the social worker in facilitating further input, discussion and referral to appropriate support groups has been invaluable. The early use of education, medication and neuropsychological input has all been beneficial. 

The majority of individuals had a good outcome using E-GOS (52%). This compares favorably to landmark studies which range from 44 - 49% (17, 32, 33).

## 5. Disscusion

Clearly these studies were carried out in different populations but we would not expect there to be much difference in baseline demographics. These results certainly encourage us that the pathway is an effective way of treating head injury patients. We hope to continue to follow up this group over time but head injury studies suffer from very high attrition rates and it will be difficult to show clear proof that the pathway has improved a hard outcome measure such as E-GOS.

One important outcome for local services has been the improvement of clinical governance with pathway responsibility and care decisions. This may be reflected in the patient and family feedback forms given to 125 patients and 125 relatives in the cohort. Replies were received from 104 patients and 97 relatives. Patients’ ratings scored an average of 4.8/5 on overall satisfaction with the service and relatives rated the service at 4.7/5. Such data is not always the most reliable outcome measurements but these patient rated outcome measures (PROMS) are becoming increasingly important as a service outcome (34).

To the best of our knowledge, we do not know of any similar RM service in the United Kingdom up to date but we know that other units are now looking to develop similar programs after discussion with us. We suggest that this pathway may be a future model that RM professionals could see to provide better care to individuals with brain injury and their families. It is also an opportunity for RM to enhance and extend its role in healthcare and improve clinical governance within health organizations. It would be interesting to know the experience of other healthcare professionals, particularly in other countries as to whether such pathways already exist and if they do, have outcomes been affected.
